# Metabolic reprogramming and transcriptomic adaptation contribute to glyphosate resistance in potato cultivars

**DOI:** 10.3389/fpls.2026.1757471

**Published:** 2026-02-12

**Authors:** Chunfang Xiao, Jianhua Gao, Yuting Zeng, Ying Zou, Zhen Wang, Denghong Zhang, Lei Yan, Guocai Yang, Yanfen Shen

**Affiliations:** 1 China Southern Potato Research Center, Enshi, Hubei, China; 2 Enshi Tujia and Miao Autonomous Prefecture Academy of Agricultural Sciences, Enshi, Hubei, China; 3 Vegetable Research Institute, Tibet Academy of Agricultural and Animal Husbandry Sciences, Lhasa, Tibet, China

**Keywords:** glyphosate resistance, metabolic reprogramming, phenylpropanoid pathway, potato, secondary metabolism, transcriptomic adaptation

## Abstract

Glyphosate, a widely used herbicide, inhibits 5-enolpyruvylshikimate-3-phosphate synthase in the shikimate pathway, and its repeated application has led to resistance in several crops, including potato. In this study, we investigated the molecular and metabolic mechanisms underlying glyphosate resistance in two contrasting potato cultivars, DP (glyphosate-tolerant) and MA (glyphosate-sensitive), using integrated transcriptomic and metabolomic analyses. Glyphosate treatment triggered cultivar-specific responses: although both cultivars activated early stress-related pathways, DP exhibited a more coordinated and sustained transcriptional response, particularly in pathways associated with detoxification, redox homeostasis, and energy regulation, whereas MA showed a broader but less organized response mainly enriched in photosynthesis and carbohydrate metabolism. Metabolomic analysis revealed pronounced metabolic reprogramming in DP, including enhanced flux through the shikimate and phenylpropanoid pathways and increased accumulation of tyrosine, ferulic acid, and flavonoids, which contribute to oxidative stress mitigation and structural defense. In contrast, MA displayed weaker metabolic adjustments, especially in secondary metabolism. Overall, these results demonstrate that glyphosate resistance in potato is driven by transcriptional plasticity and metabolic reprogramming that enhance secondary metabolism and stress tolerance, providing new insights into herbicide resistance mechanisms.

## Introduction

Potato (*Solanum tuberosum* L.), the world’s third most important food crop, provides a significant source of carbohydrates and plays a pivotal role in global food security. In China, the largest potato producer globally, it supports essential food industries, contributes to rural development, and improves farmers’ incomes, especially in less developed regions (Wang et al., 2023; Waaswa et al., 2021). Despite its importance, early-stage potato cultivation is threatened by weed competition, necessitating the use of herbicides such as glyphosate. While glyphosate is highly effective, excessive use can cause crop phytotoxicity and environmental impacts, emphasizing the need for herbicide-tolerant potato varieties to promote sustainable agriculture (Adeux et al., 2019).

Glyphosate, introduced in, 1974, acts as a non-selective herbicide by inhibiting 5-enolpyruvylshikimate-3-phosphate synthase (EPSPS), blocking the shikimate pathway, and thereby reducing the biosynthesis of aromatic amino acids (phenylalanine, tyrosine, and tryptophan) essential for protein synthesis and secondary metabolism (Duke and Powles, 2008; Maeda and Dudareva, 2012; Gaines et al., 2020). Its widespread use has led to the emergence of glyphosate-resistant weeds and raised environmental concerns, including disruptions to soil microbial communities and reduced nitrogen fixation (Délye et al., 2013; Duke et al., 2012; Helander et al., 2012; Newman et al., 2016; Van Bruggen et al., 2018). Despite these issues, glyphosate remains critical in no-till farming systems, achieving effective control of deep-rooted perennial weeds such as *Sorghum halepense* (Shaner, 2009; Vila-Aiub et al., 2012; Benbrook, 2016).

Glyphosate resistance in crops and weeds has become an increasingly significant issue in agriculture. Multiple studies have explored the molecular mechanisms underlying this resistance. Wang et al. (2024) identified potential genes associated with glyphosate resistance in cassava through transcriptomic analysis, offering insights into the metabolic pathways that could be targeted for improved resistance. Similarly, Guo et al. (2021) explored the differential responses of EPSPS and GAT genes in transgenic soybeans, uncovering tolerance mechanisms that may be useful for future crop management strategies. In the case of *Amaranthus palmeri*, Fernández-Escalada et al. (2016) observed differences in oxidative stress responses between resistant and susceptible populations, suggesting that enhanced antioxidant activity contributes to glyphosate tolerance. Other studies, such as those by Ahsan et al. (2008) and Maroli et al. (2015), have emphasized the role of oxidative stress in glyphosate resistance, showing that resistant biotypes exhibit increased antioxidant levels to mitigate the herbicide’s toxic effects. Research on glyphosate-resistant species, including *Lolium multiflorum* (Cechin et al., 2020) and *Amaranthus tuberculatus* (Lorentz et al., 2014), has revealed key genetic contributors to glyphosate resistance, such as EPSPS mutations and altered herbicide transport mechanisms. Collectively, these studies demonstrate that gene expression profiling not only facilitates the identification of candidate genes associated with glyphosate resistance, but also provides critical mechanistic insights into resistance pathways. Such knowledge supports resistance diagnostics, improves the prediction of potential cross-resistance, and informs the development of integrated weed management strategies aimed at mitigating resistance evolution and ensuring long-term agricultural productivity.

The development of glyphosate-tolerant potato cultivars offers a practical solution for effective weed management while supporting environmental sustainability. Such varieties can mitigate the risks of phytotoxicity, reduce reliance on alternative herbicides, and facilitate integrated weed management. Integrated multi-omics approaches, combining genomics, transcriptomics, metabolomics, and phenotyping, provide a powerful framework to dissect complex stress responses and identify key regulatory networks. Despite progress in transgenic strategies, such as EPSPS overexpression, the molecular basis of non-transgenic glyphosate tolerance in potato remains poorly understood. Understanding mechanisms including detoxification pathways, antioxidant regulation, and metabolic adaptations in tolerant genotypes is essential for precise breeding. In this study, we apply a multi-omics approach to contrasting tolerant potato genotypes, aiming to elucidate their metabolic and genetic mechanisms of glyphosate tolerance and to identify actionable targets for the development of next-generation herbicide-resistant cultivars.

## Materials and methods

### Plant materials, glyphosate treatment and sample collection

Two potato (*Solanum tuberosum* L.) cultivars, De Shu No.3 (DP) and Mira (MA), were selected, of which DP is a glyphosate-tolerant cultivar and MA is a glyphosate-sensitive cultivar that was introduced from Germany in, 1956 and widely cultivated in China. The potato varieties are all derived from the Potato Germplasm Resource Bank of Hubei Province and provided by the China Southern Potato Research Center in Enshi, Hubei. Virus-free seed tubers were germinated at 20°C until sprouts reached ~1 cm. Sprouted tubers were planted in pots containing a vermiculite-nutrient soil mixture (one tuber per pot) and cultivated in a growth chamber under controlled conditions: 22°C, 70% relative humidity, and a 16 h light/8 h dark photoperiod. Five-week-old seedlings were treated with glyphosate via foliar spraying. A commercial glyphosate formulation (41% isopropylamine salt, corresponding to 30% glyphosate acid equivalent; Roundup^®^, Monsanto, USA) was applied at a rate of 3 mL formulation per liter of water (1:333 dilution), resulting in a final glyphosate acid-equivalent concentration of 0.9 g L⁻¹. Leaf samples were collected at four-time points: 0 d (T1, control), 1 d (T2), 3 d (T3), and 5 d (T4) post-treatment. For each cultivar and time point, three biological replicates were collected, with each replicate consisting of three upper fully expanded leaves. All samples were immediately frozen in liquid nitrogen and stored at −80°C until analysis.

### RNA extraction and sequencing

Total RNA was extracted from 100 mg leaf tissue using the RNAprep Pure Plant Kit (Tiangen Biotech, China). RNA integrity was verified by agarose gel electrophoresis and quantified using a NanoDrop, 2000 spectrophotometer (Thermo Fisher Scientific, USA). RNA-seq libraries were constructed with the NEBNext Ultra II RNA Library Prep Kit (NEB, USA) and sequenced on an Illumina NovaSeq, 6000 platform (PE150 mode), generating ~6 Gb raw data per sample. Biological replicates were randomized during library preparation and sequencing. Samples from different experimental groups were processed simultaneously and distributed across sequencing runs to avoid systematic effects.

### Transcriptome data analysis

Raw reads were quality-filtered using fastp (v0.23.2) to remove adapters and low-quality sequences (Phred score < 20). Clean reads were aligned to the *S. tuberosum* reference genome (Assembly: SolTub_3.0) using HISAT2 (v2.2.1) with default parameters. Gene expression quantification was performed with featureCounts (v2.0.3) based on uniquely mapped reads. Differentially expressed analysis was performed using DESeq2 with default dispersion estimation procedures. P-values were adjusted for multiple testing using the Benjamini–Hochberg method, and genes with an adjusted p-value (FDR) < 0.05 were considered significantly differentially expressed. Differentially expressed genes (DEGs) were finally filtered with |log2(fold change)| ≥1 and adjusted p-value threshold of 0.05. Functional annotation of DEGs was conducted using Gene Ontology (GO), Kyoto Encyclopedia of Genes and Genomes (KEGG), and online software g:Profiler was applied to conduct enrichment analysis of DEGs.

### Preparation of metabolic samples

For each sample, 100 mg of powder was weighed and mixed with 1.0 mL of 70% methanol containing 0.1 mg/L acyclovir as an internal standard. The mixture was vortexed and ultrasonicated for 15 minutes, followed by overnight extraction at 4°C. After centrifugation at 14000 × g for 10 minutes, the supernatant was filtered through a 0.22-µm nylon filter suitable for organic solvents (SCAA-104; ANPEL, China) before LC–MS analysis. Additionally, aliquots from each sample were pooled to create quality control (QC) samples for monitoring instrument performance.

### Metabolites detection

Metabolite profiling was conducted using an ExionLC UPLC instrument coupled with a 5500+ QTRAP mass spectrometer (AB SCIEX, Foster City, CA), equipped with an electrospray ionization (ESI) source. The mobile phases consisted of solvent A (water with 0.04% acetic acid and solvent B (acetonitrile with 0.04% acetic acid). The gradient program was as follows: 95:5 (A:B) at 0 min, transitioning to 5:95 at 10 min, held for 3 min, returning to 95:5 at 13.1 min, and maintained for 2.9 min. The column oven was maintained at 40°C, with a flow rate of 0.35 mL/min and an injection volume of 5 µL. Instrument control, data acquisition, and processing were carried out using Analyst 1.7.2 software (AB SCIEX). Separation was achieved on an ACQUITY PRM HSST3 column (130 A˚, 1.8 µm, 2.1 mm × 100 mm). The optimized ESI operating parameters for negative mode were: IS, -4500 V; CUR, 40 psi; TEM, 450°C; GS1, 40 psi; GS2, 40 psi. For positive mode, they were: IS, 5500 V; CUR, 40 psi; TEM, 550°C; GS1, 40 psi; GS2, 40 psi. Scheduled multiple reaction monitoring (sMRM) was used for metabolite detection, with a 60-second MRM detection window and 1.0-second target scan time. Chromatographic peaks were integrated and corrected using MultiQuant OS (AB SCIEX, Concord, ON, Canada), and peak area integrals were used to quantify relative abundance of metabolites.

### Statistical analysis

Metabolite data were unit variance–scaled prior to principal component analysis (PCA), which was performed using the *FactoMineR* R package to visualize overall metabolic variation among samples. Hierarchical clustering analysis was conducted using the *pheatmap* R package based on Euclidean distance. For orthogonal partial least squares–discriminant analysis (OPLS-DA), metabolite data were log_2_-transformed and mean-centered, and supervised models were constructed to discriminate between treatment groups within each cultivar at individual time points. OPLS-DA score plots and permutation tests were generated using the *MetaboAnalystR* package (Pang et al., 2024) to evaluate model robustness and avoid overfitting. To account for temporal variation, metabolomic comparisons were conducted separately for each sampling time point, allowing the identification of time-specific metabolic responses to glyphosate treatment without assuming uniform temporal trends. Differentially accumulated metabolites were identified based on variable importance in projection (VIP) scores from OPLS-DA (VIP > 1.0) combined with absolute log_2_ fold change thresholds. Where applicable, statistical significance was further assessed using univariate analyses with p-values adjusted for multiple testing. Volcano plots and Venn diagrams were generated using the ggplot2 package (Wickham, 2009). Identified metabolites were annotated using the KEGG Compound database, and pathway enrichment analysis was performed using hypergeometric testing to identify significantly affected metabolic pathways.

## Results

### Phenotypic responses of potato cultivars to glyphosate treatment

**Figure 1:**
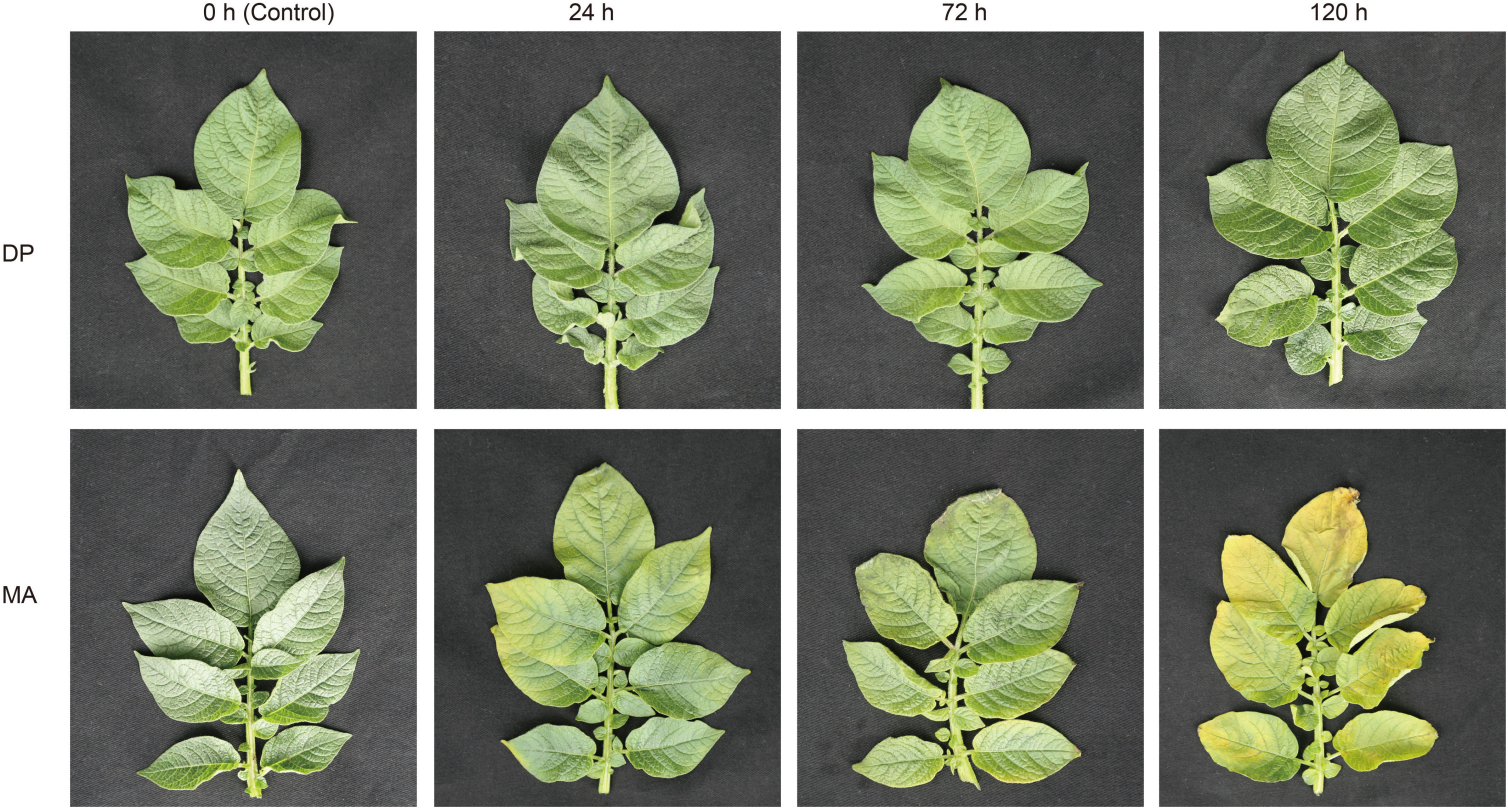
Phenotypic differences between DP and MA following glyphosate treatment. DP refers to the glyphosate-tolerant cultivar De Shu No.3, and MA refers to the glyphosate-sensitive cultivar Mira.

To evaluate the phenotypic impact of glyphosate, leaves from five-week-old plants of the potato cultivars DP and MA were treated with glyphosate (**Figure 1**). At 0 h (control), all leaves appeared uniformly fresh green, reflecting healthy growth conditions. Upon glyphosate exposure, MA leaves exhibited progressive chlorosis, initiating at the leaf base and extending toward the margins. By 72 h post-treatment, distinct necrotic lesions had developed on MA leaves, and by 120 h, widespread yellowing affected the majority of the leaf surface. In contrast, DP leaves exhibited mild and uniform chlorosis without necrosis, even at 120 h. These observations demonstrate that DP has a higher tolerance to glyphosate, while MA is more susceptible.

### Gene expression dynamics in tolerant and sensitive cultivars under glyphosate stress

**Figure 2:**
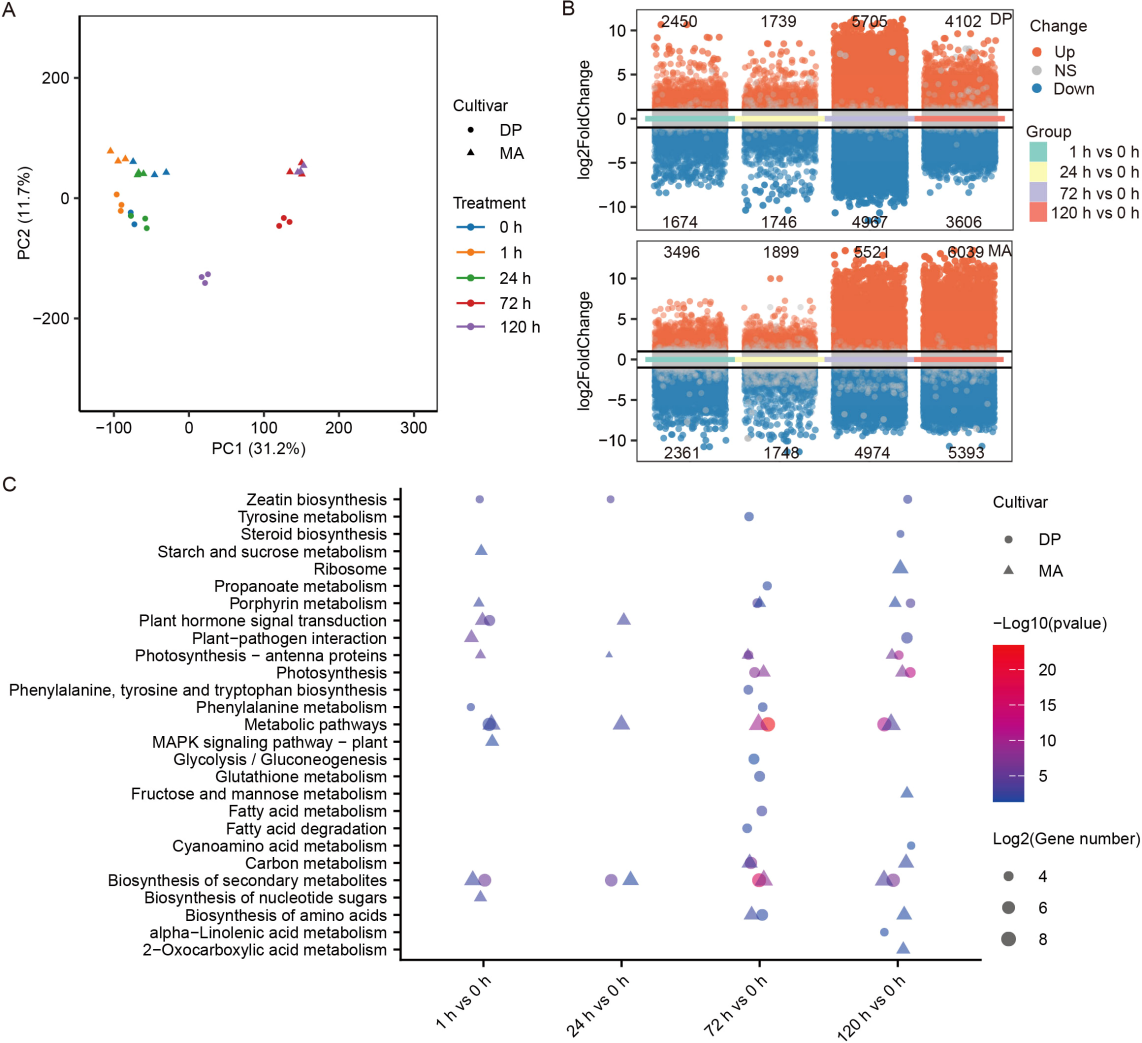
Transcriptomic profiling of potato leaves under glyphosate stress. **(A)** Principal component analysis (PCA) illustrating transcriptomic variation in DP and MA before and after glyphosate treatment. **(B)** Volcano plots depicting the number and distribution of differentially expressed genes (DEGs) across comparisons. **(C)** Bubble plots representing KEGG pathway enrichment analysis of DEGs in each comparison.

To investigate gene expression dynamics in DP and MA under glyphosate stress, samples collected before and after treatment were subjected to RNA sequencing. In total, 30 samples generated high-quality transcriptomic data (**Supplementary Table S1**), with 39.6–63.2 million reads per sample. All libraries exhibited excellent sequencing quality, with Q20 and Q30 scores above 99% and 97%, respectively, GC content ranging from 41.1% to 44.9%, and mapping rates between 80.9% and 89.3%, indicating the reliability of the data for subsequent analyses. A total of 21, 476 protein-coding genes were detected (**Supplementary Table S2**), with 78.6% exhibiting TPM > 1 in at least one sample. Principal component analysis (PCA) revealed clear clustering of biological replicates, with PC1 and PC2 explaining 31.2% and 11.7% of variance, respectively (**Figure 2A**). Temporal dispersion increased from 0 h to 120 h, indicating progressive transcriptional reprogramming. Samples at 1 h clustered near the control, while samples at 24 h, 72 h, and 120 h diverged significantly.

Differentially expressed genes (DEGs) showed dynamic variation across time points and between genotypes (**Figure 2B**; **Supplementary Table S2**). At 1 h post-treatment, DP exhibited 2, 450 upregulated and 1, 674 downregulated genes, whereas MA had 3, 496 upregulated and 2, 361 downregulated genes. The magnitude and direction of gene expression changed dynamically over 120 h, revealing genotype-specific transcriptomic responses to glyphosate stress.

### Functional enrichment of DEGs following glyphosate exposure

Gene ontology (GO) analysis showed both genotypes were enriched stress-related categories, including response to stimulus and phosphorylation, at early time points (1 h, 24 h), indicating initial activation of general defense mechanisms (**Supplementary Figure S1**). However, marked genotype-specific differences were observed in both the temporal dynamics and functional categories of enriched pathways. The DP cultivar demonstrated pronounced enrichment in carbohydrate metabolic process, organophosphate metabolic process, and defense response pathways at later time points (72 h and 120 h), indicating a robust and sustained transcriptional reprogramming contributing to its glyphosate tolerance. In contrast, the MA cultivar showed enrichment predominantly in photosynthesis-related and phosphorylation processes, particularly at 120 h, suggesting a prolonged disruption of primary metabolic functions under glyphosate stress. Notably, pathways such as response to hydrogen peroxide, lipid transport, defense response, phenylpropanoid metabolic process, and carboxylic acid metabolism were more strongly or earlier activated in DP, reflecting more efficient stress responses, nutrient utilization, and energy adaptation mechanisms compared to the sensitive MA genotype.

KEGG pathway enrichment analysis revealed both distinct and overlapping transcriptional responses to glyphosate treatment in DP and MA cultivars (**Figure 2C**). In both cultivars, the enriched pathways predominantly involved metabolic processes and stress-response signaling. Plant hormone signal transduction and secondary metabolites biosynthesis were enriched in both genotypes after 1 h glyphosate treatment, reflecting conserved early stress signaling. However, several pathways such as starch and sucrose metabolism, porphyrin metabolism, MAPK signaling pathway–plant, and biosynthesis of nucleotide sugars were enriched only in MA after 1 h and 24 h glyphosate treatment whereas phenylalanine metabolism was only enriched in DP after 1 h glyphosate treatment. At 72 h and 120 h, DP exhibited sustained activation of fatty acid metabolism, glutathione metabolism, and biosynthesis of secondary metabolites, particularly, suggesting stronger detoxification and metabolic adaptation. In contrast, MA showed more pronounced enrichment in porphyrin metabolism, tyrosine metabolism, and starch and sucrose metabolism, which may indicate stress-related metabolic shifts linked to sensitivity. These results demonstrate genotype-specific temporal dynamics in glyphosate stress adaptation and cellular reprogramming.

### Cultivar-specific gene expression patterns in response to glyphosate

**Figure 3:**
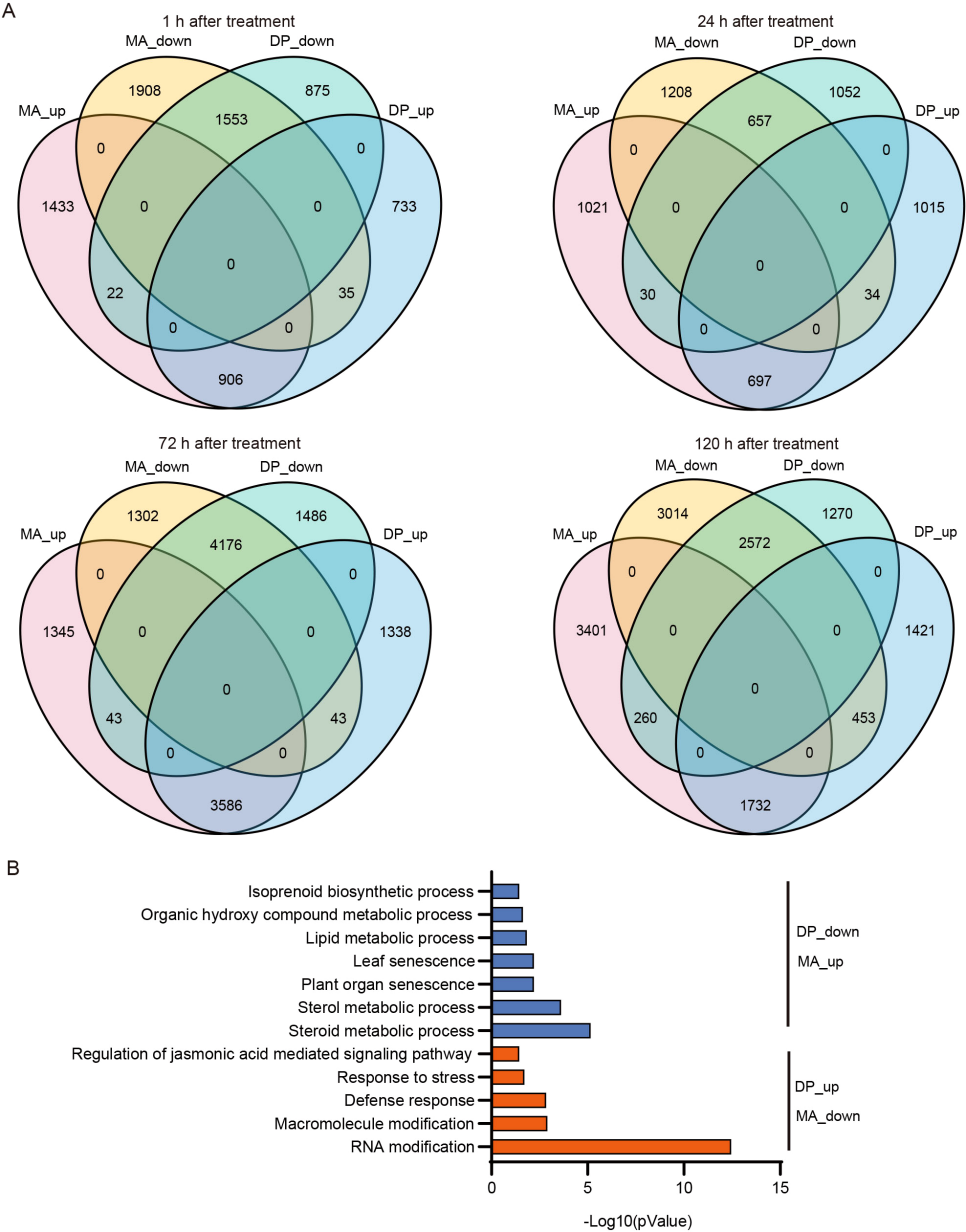
Genotype-specific transcriptional responses to glyphosate treatment. **(A)** Venn diagrams showing the number of DEGs in DP and MA at each time point post-treatment. **(B)** Gene Ontology (GO) enrichment analysis highlighting divergent functional responses in the two cultivars.

Comparative DEG analysis revealed both shared and unique expression profiles between cultivars across different time points (**Figure 3A**). At 1 h, MA and DP showed 1, 433 and 733 uniquely upregulated genes, respectively, with 1, 553 shared. By 120 h, MA displayed 3, 401 uniquely upregulated genes versus 1, 421 in DP. A similar trend was observed for downregulated genes. Notably, a subset of DEGs (57 at 1 h, increasing to 713 at 120 h) showed opposing expression patterns between cultivars, representing candidates for functional divergence. GO enrichment of these opposing DEGs indicated that genes upregulated in DP but downregulated in MA were involved in RNA modification, defense response (such as *PGSC0003DMG401024548*, *PGSC0003DMG400039376*, *PGSC0003DMG400004295*, *PGSC0003DMG401021043*), and jasmonic acid signaling (such as *PGSC0003DMG400001178*, *PGSC0003DMG401000923*, *PGSC0003DMG400008781*, *PGSC0003DMG400002930*, *PGSC0003DMG400022888*). Conversely, genes downregulated in DP and upregulated in MA were enriched in steroid metabolism, leaf senescence, and isoprenoid biosynthesis (such as *PGSC0003DMG400004253*, *PGSC0003DMG400027856*, *PGSC0003DMG402031070*, *PGSC0003DMG401031070*, *PGSC0003DMG400018679*) (**Figure 3B**). These findings highlight critical regulatory nodes differentiating tolerance and sensitivity.

Detoxification is a key physiological process in response to glyphosate exposure, with peroxidases (PODs), ATP-binding cassette (ABC) transporters, and UDP-glycosyltransferases playing critical roles in this mechanism. A substantial number of genes encoding these protein families were differentially expressed in both DP and MA cultivars. A total of 45 POD genes were identified as differentially expressed; although the majority were downregulated in response to glyphosate treatment, DP exhibited a greater number of specifically induced and upregulated PODs during the early stages (**Supplementary Figure S2**). Eighty ABC transporter genes were identified as differentially expressed, suggesting that glyphosate-induced stress activates this transporter family. Most of these genes (e.g., *PGSC0003DMG400023506*) were upregulated after 72 hours in both cultivars, with MA showing a more pronounced transcriptional response (**Supplementary Figure S3**). Similarly, 139 UDP-glycosyltransferase genes were differentially expressed, as with ABC transporters, the magnitude of expression changes in MA was generally higher than in DP (e.g., *PGSC0003DMG400017249*), indicating a genotype-specific detoxification response pattern. These differential expression patterns of genes encoding PODs, ABC transporters, and UDP-glycosyltransferases provide valuable insights into the distinct detoxification strategies employed by the DP and MA cultivars in response to glyphosate exposure.

### Metabolic reprogramming in response to glyphosate treatment

**Figure 4:**
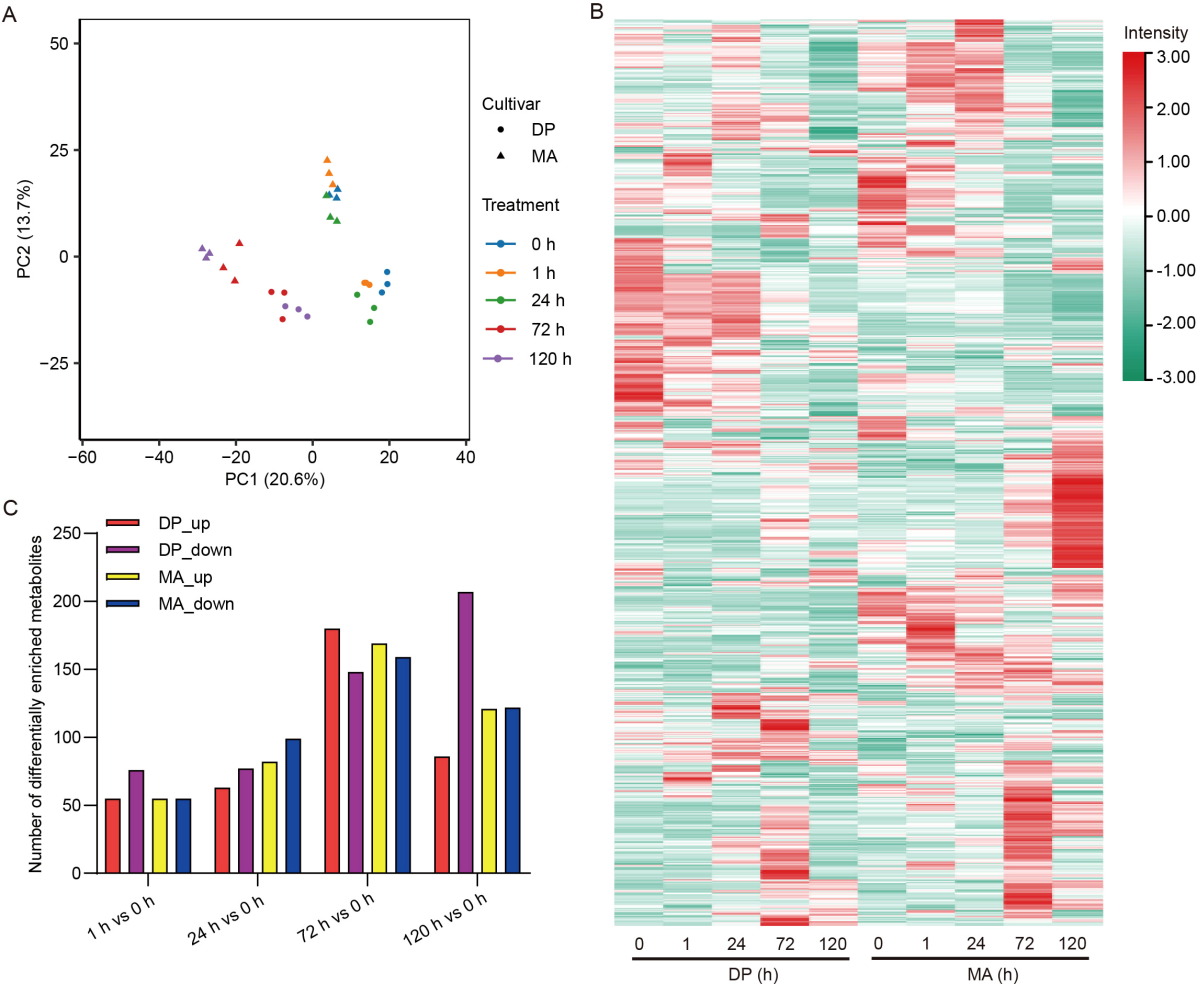
Metabolomic responses to glyphosate treatment. **(A)** PCA of metabolite profiles in DP and MA before and after glyphosate exposure. **(B)** Heatmap illustrating temporal metabolite changes in DP and MA. Rows represent metabolites clustered by unsupervised hierarchical clustering based on their accumulation patterns, and columns represent samples. Color gradients reflect normalized abundance, with red indicating upregulation and green indicating downregulation. **(C)** Bar plots showing the number of differentially accumulated metabolites (DAMs), categorized as upregulated (DP_up, MA_up) or downregulated (DP_down, MA_down) based on log_2_(FoldChange) > 1 or < -1 and VIP > 1.

A total of 1, 042 metabolites were quantified. Principal component analysis (PCA) using all metabolites revealed partial separation between DP and MA along PC1 (~20.6% variance) (**Figure 4A**), indicating inherent metabolic differences between the two cultivars. Sample clustering shifts progressively farther from the origin with increasing treatment duration (e.g., 0 h vs. 120 h), suggesting cumulative metabolic changes over time due to divergent adaptive responses or metabolic saturation. Additionally, the overlap between 0 h and 1 h samples in both cultivars suggests minimal immediate perturbation, whereas treatments from 24 to 120 h result in more pronounced metabolic shifts. This pattern is consistent with a delayed activation of metabolic pathways in response to stress.

Distinct temporal metabolic dynamics were observed within each cultivar (**Figure 4B**). In both DP and MA, early time points (0 h and 1 h) showed relatively modest alterations, indicating minimal immediate metabolic disturbance. More pronounced shifts in metabolite intensity emerged from 24 h onward, with significant changes occurring at 72 h and 120 h, reflecting progressive and cumulative metabolic reprogramming in response to glyphosate treatment. Notably, MA exhibited broader and more intense changes in metabolite profiles at later time points compared to DP, suggesting genotype-specific differences in metabolic adaptation and stress response. These temporal patterns support the hypothesis of divergent metabolic trajectories and underlying regulatory mechanisms between the two cultivars under prolonged herbicide exposure.

A time-dependent increase in differentially accumulated metabolites was observed in both cultivars, with the most significant changes occurring at 72 h and 120 h post-treatment (**Figure 4C**). At 1 h and 24 h, relatively few metabolites showed significant enrichment, suggesting a limited early metabolic response. However, by 72 h, the number of differentially accumulated metabolites increased markedly, particularly in DP, which displayed a higher proportion of upregulated metabolites relative to MA. At 120 h, DP exhibited a notable increase in downregulated metabolites, whereas MA exhibited a more even distribution between up- and downregulated compounds. These results indicate a delayed yet robust metabolic reprogramming in response to glyphosate, with cultivar-specific differences in both the extent and direction of metabolic changes. The increased downregulated of metabolites in DP at 120 h may indicate metabolic suppression or resource reallocation under stress, in contrast to the relatively stabilized metabolic profile observed in MA.

### Differentially accumulated metabolites between two cultivars

**Figure 5:**
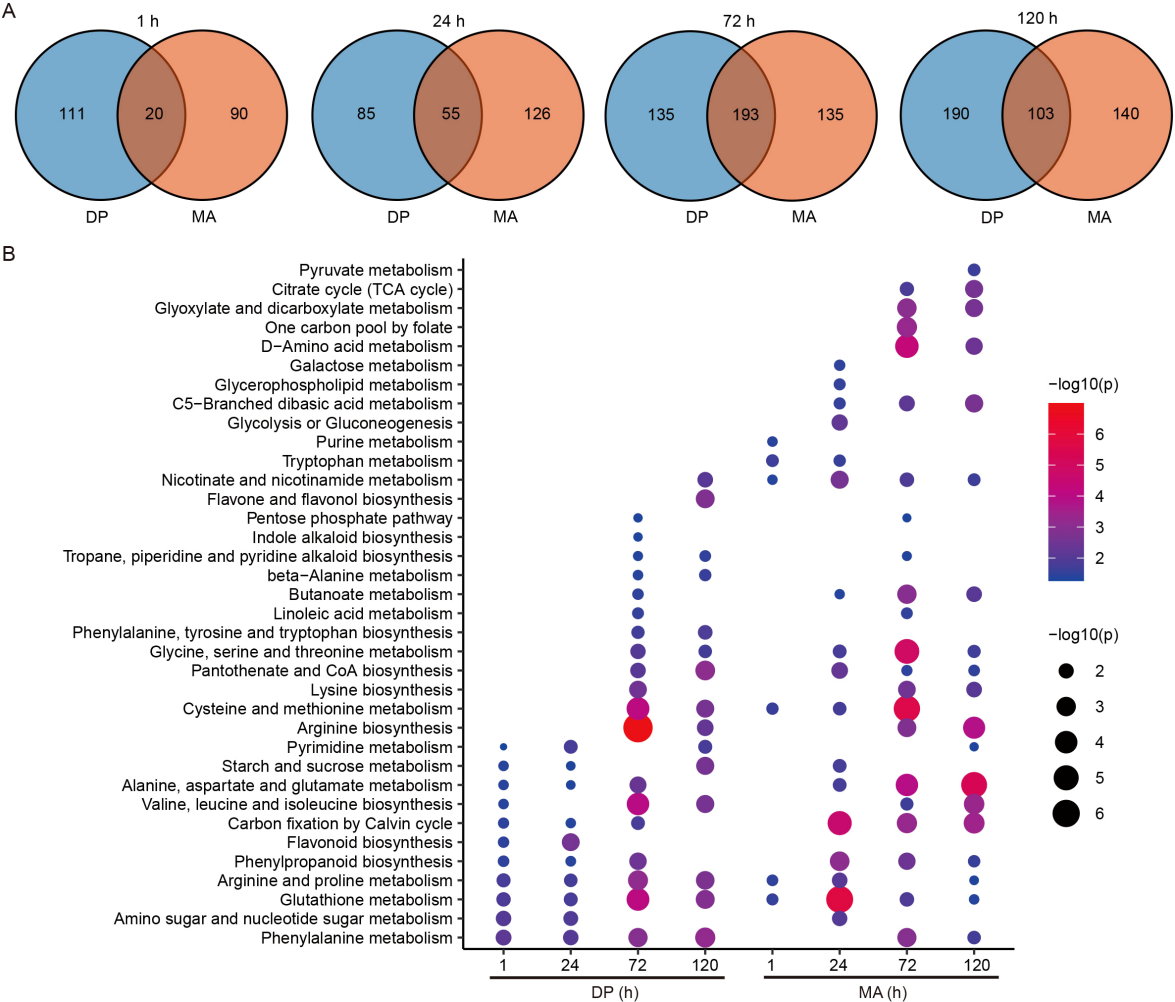
Comparative metabolomic dynamics between DP and MA. **(A)** Venn diagrams depicting the number of DAMs unique to or shared between DP and MA across four time points post-glyphosate treatment. **(B)** Metabolic pathway enrichment analysis of DAMs in each comparison group.

The temporal dynamics of differentially accumulated metabolites (DAMs) were examined in DP and MA following glyphosate treatment (**Figure 5A**). Venn diagram analysis revealed distinct genotype-specific and time-dependent responses. At 1 h post-treatment, DP and MA exhibited 111 and 90 unique DAMs, respectively, with only 20 shared DAMs, indicating an early divergent metabolic response. By 24 h, shared DAMs increased to 55, suggesting partial convergence of metabolic activity. At 72 h, a marked overlap of 193 shared DAMs was observed, with an equal number of unique DAMs (135) in both genotypes, reflecting a strong common metabolic adaptation to herbicide stress. At 120 h, although shared DAMs declined to 103, both genotypes exhibited a high number of unique DAMs (DP: 190; MA: 140), indicating sustained yet divergent metabolic reprogramming. These results underscore the dynamic and genotype-specific nature of the metabolic response to glyphosate over time. Pathway enrichment analysis further highlighted temporal metabolic divergence between DP and MA (**Figure 5B**). MA showed significant enrichment in pathways associated with energy metabolism and amino acid biosynthesis, including pyruvate metabolism, the TCA cycle, and glycolysis/gluconeogenesis. In contrast, DP demonstrated stronger enrichment in secondary metabolic pathways such as phenylpropanoid biosynthesis and flavonoid/flavonol biosynthesis. Notably, the arginine biosynthesis pathway was persistently enriched in DP after 72 h, suggesting its involvement in stress adaptation. These findings reveal fundamental differences in energy utilization strategies and redox balance mechanisms between the two cultivars under glyphosate-induced stress.

### Dynamic regulation of shikimate pathway

**Figure 6:**
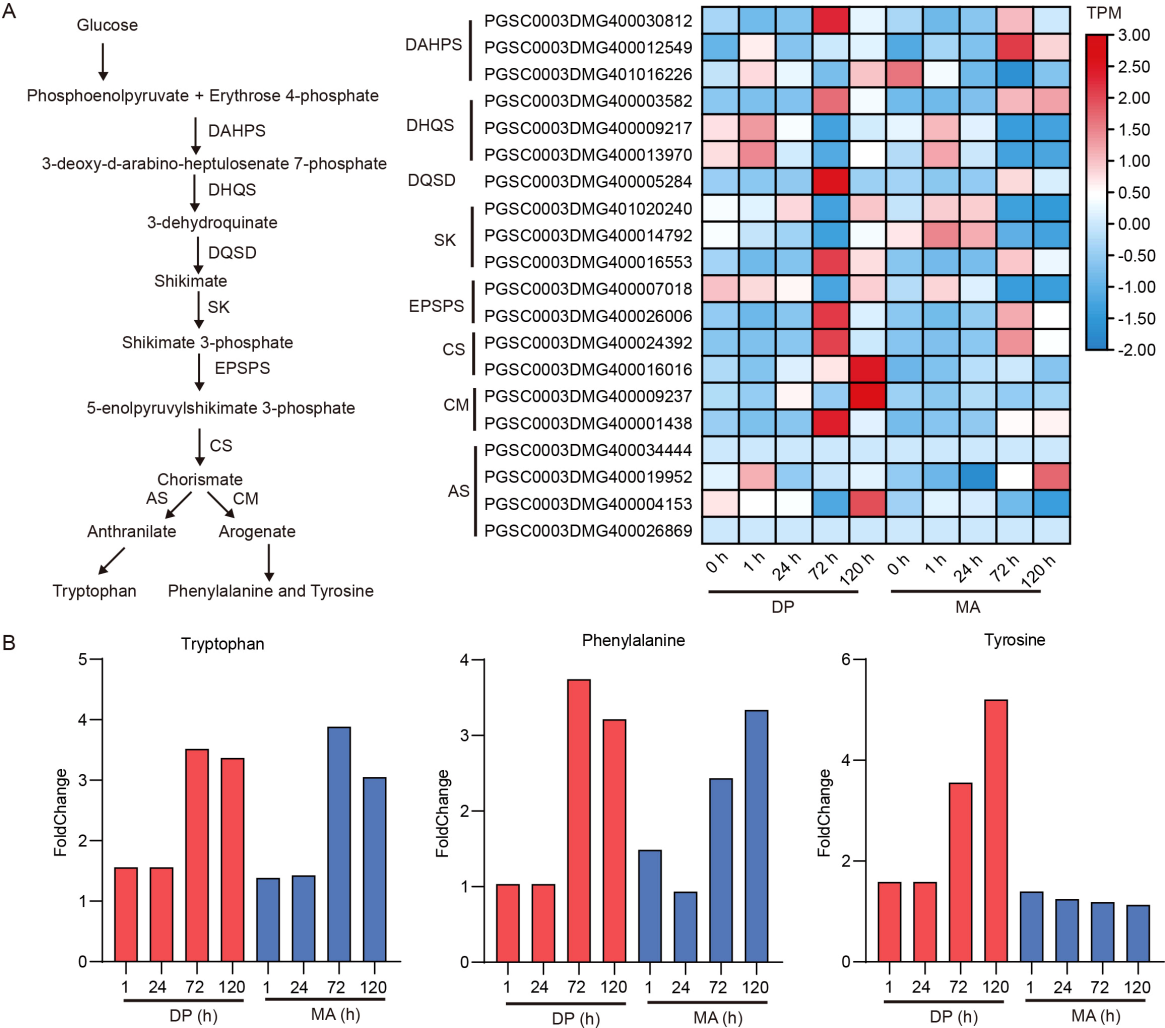
Gene expression and metabolite dynamics within the shikimate pathway. **(A)** Expression profiles of key enzymes in the shikimate pathway. Abbreviations: DAHPS, D-arabino-heptulosonate 7-phosphate synthase; DHQS, dehydroquinate synthase; DQSD, 3-dehydroquinate dehydratase/shikimate dehydrogenase; SK, shikimate kinase; EPSPS, 5-enolpyruvylshikimate-3-phosphate synthase; CS, chorismate synthase; CM, chorismate mutase; AS, anthranilate synthase. **(B)** Bar charts showing fold changes of tryptophan, phenylalanine, and tyrosine under glyphosate treatment across four time points.

Glyphosate treatment targets the 5-enolpyruvylshikimate-3-phosphate synthase (EPSPS) enzyme, a key component of the shikimate pathway. Comparative gene expression analysis revealed distinct regulatory responses between DP and MA (**Figure 6A**). In DP, genes encoding DAHPS (e.g., *PGSC0003DMG400030812*, *PGSC0003DMG401016226*) and EPSPS (*PGSC0003DMG400026006*) exhibited sustained upregulation following glyphosate exposure. Early induction of DHQS and DQSD was also observed, suggesting enhanced flux toward aromatic amino acid biosynthesis. In contrast, MA displayed transient induction, peaking at 24 h but failing to sustain elevated expression. EPSPS expression remained consistently higher in DP, especially at early stages (1–24 h), likely contributing to increased production of shikimate-3-phosphate. Upregulation of downstream enzymes such as CS (*PGSC0003DMG400024392*), CM (*PGSC0003DMG400001438*), and AS (*PGSC0003DMG400004153*) in DP further supports efficient precursor synthesis for downstream metabolic processes. Metabolite quantification of tryptophan, phenylalanine, and tyrosine revealed genotype-specific differences in response to glyphosate (**Figure 6B**). While tryptophan and phenylalanine levels increased slightly at 1 h and 24 h in both cultivars, tyrosine showed the most dramatic variation. DP exhibited an approximately ~4-fold increase in tyrosine accumulation at 72 h, compared to minor fluctuations (<1.5-fold) in MA. These results indicate that glyphosate treatment primarily affects tyrosine biosynthesis, with DP displaying a more robust metabolic adaptation.

### Differential accumulation of metabolites in the phenylpropanoid pathway

**Figure 7:**
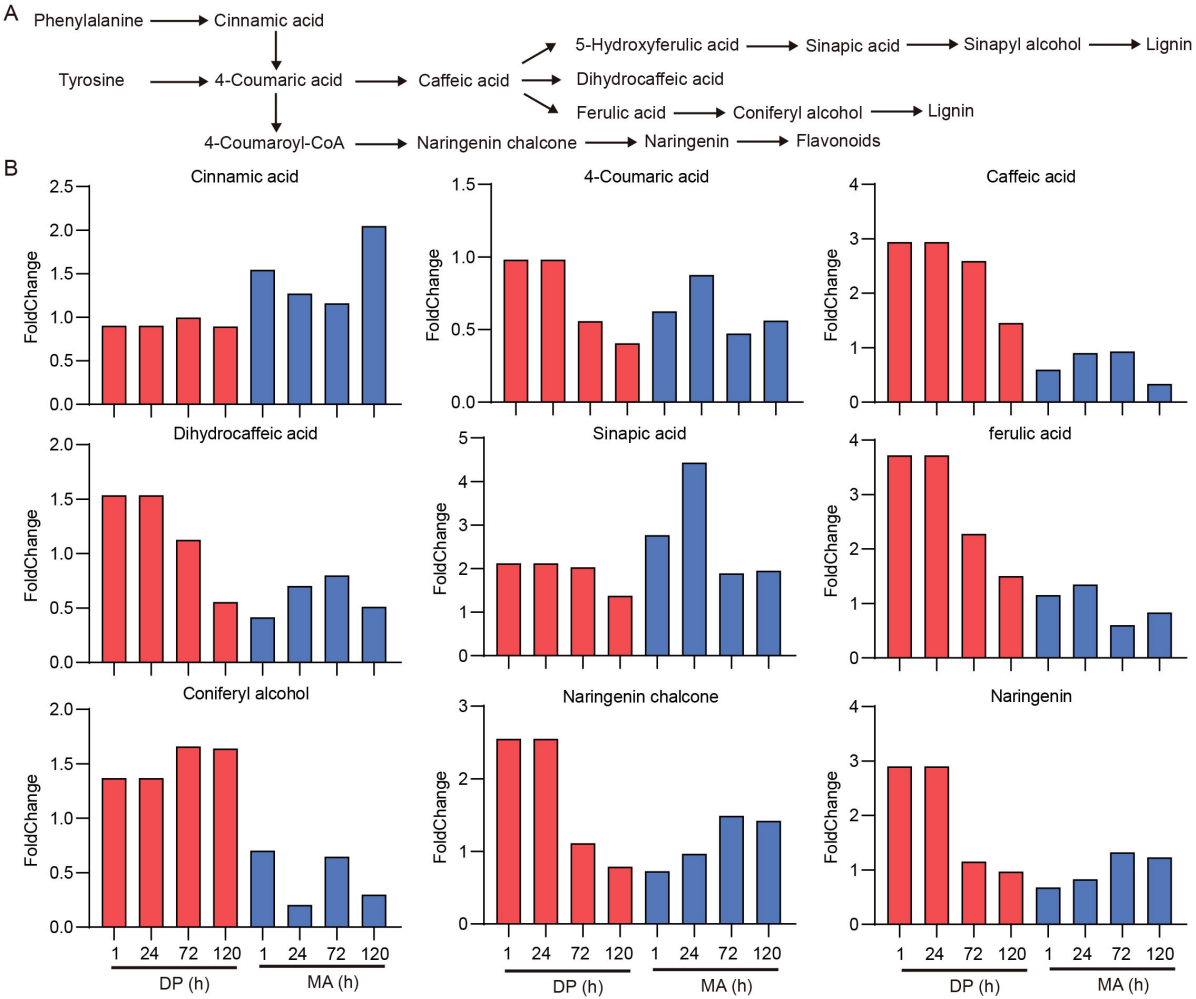
Metabolite changes in the phenylpropanoid pathway in response to glyphosate. **(A)** Schematic diagram of the phenylpropanoid metabolic pathway involved in lignin and flavonoid biosynthesis and stress response. **(B)** Fold changes of key intermediates and end products involved in lignin and flavonoid biosynthesis following glyphosate exposure.

Phenylalanine and tyrosine serve as precursors for lignin and flavonoid biosynthesis, with intermediates such as 4-coumaric acid, caffeic acid, ferulic acid, sinapyl alcohol, and coniferyl alcohol playing critical roles (**Figure 7A**). Given the distinct regulation of tyrosine under glyphosate stress, several metabolites associated with the downstream phenylpropanoid pathway exhibited differential responses between the two cultivars (**Figure 7B**). Cinnamic acid levels remained unchanged in DP and were slightly increased in MA, whereas 4-coumaric acid was downregulated in both cultivars. In contrast, caffeic acid, dihydrocaffeic acid, ferulic acid, and coniferyl alcohol showed pronounced increases in DP but only modest changes in MA. Among these, ferulic acid displayed the most notable difference, with DP showing an approximately four-fold higher response intensity at 1 h post-treatment. The reported fold changes are based on metabolite peak areas rather than absolute concentrations or fluxes. Thus, the increases at 1 h likely reflect early metabolic responsiveness associated with stress perception, rather than complete pathway-level activation. In the flavonoid branch of phenylpropanoid metabolism, DP maintained elevated levels of naringenin chalcone and naringenin following treatment. At 24 h, naringenin levels in DP were nearly 3-fold higher than in MA, which showed no significant change. Collectively, these results suggest that glyphosate resistance in DP is associated with a stronger and more sustained responsiveness of the phenylpropanoid pathway, leading to enhanced synthesis of lignin and flavonoids, which may contribute to improved stress tolerance.

## Discussion

Glyphosate is the most extensively used herbicide worldwide and exerts its phytotoxic effects primarily by inhibiting EPSPS, a key enzyme in the shikimate pathway responsible for the biosynthesis of aromatic amino acids, including phenylalanine, tyrosine, and tryptophan (Duke and Powles, 2008). Inhibition of this pathway disrupts essential metabolic processes required for plant growth and survival, ultimately leading to plant death. Extensive and repeated glyphosate application has resulted in the evolution of resistance in more than 50 weed species worldwide (http://www.weedscience.org/). Similarly, crop plants such as potato exhibit cultivar-dependent differences in glyphosate tolerance. In this study, the tolerant cultivar DP showed no visible damage at 120 h post-treatment, whereas the sensitive cultivar MA developed chlorosis as early as 24 h (**Figure 1A**), indicating substantial genotypic variation in glyphosate response. Integrated transcriptomic and metabolomic analyses indicated that this phenotypic divergence is associated with distinct regulatory patterns, including differences in the temporal dynamics and functional distribution of gene expression and metabolic responses. In particular, DP exhibited changes concentrated in pathways related to transcriptional regulation, antioxidant defense, and metabolic adjustment, whereas MA showed broader but less functionally focused alterations.

Transcriptomic profiling revealed marked differences in gene expression dynamics between the two cultivars under glyphosate stress (**Figure 2**). Both DP and MA activated early stress-responsive pathways following treatment; however, their subsequent transcriptional trajectories diverged over time. In DP, differentially expressed genes at later time points (72 h and 120 h) were consistently enriched in a limited number of functionally related pathways, including detoxification processes, redox homeostasis (e.g., glutathione metabolism), and energy-related pathways. In contrast, MA displayed transcriptional changes distributed across a wider range of pathways, with reduced enrichment consistency at later stages. Importantly, many differentially expressed genes (DEGs) exhibited genotype-specific expression patterns (**Figure 3**), particularly in jasmonic acid signaling, peroxidases (PODs), UDP-glucosyltransferases (UGTs), and ABC transporters—gene families previously implicated in herbicide detoxification and tolerance (Gaines et al., 2020; Yuan et al., 2022). These findings suggest that transcriptional control, especially the timely and selective induction of defense-related genes, is a key determinant of glyphosate resistance.

In addition to transcriptional reprogramming, metabolomic analysis revealed dynamic and cultivar-specific metabolic adaptations in response to glyphosate exposure. Principal component analysis and DAMs profiling demonstrated that both cultivars underwent time-dependent metabolic shifts, with DP showing more robust and targeted changes, particularly at 72 h and 120 h (**Figures 4**, **5**). Given that glyphosate directly inhibits EPSPS in the shikimate pathway, disrupting the biosynthesis of aromatic amino acids such as tryptophan, phenylalanine, and tyrosine (Steinrücken and Amrhein, 1980), we focused on pathway-specific responses. In DP, sustained upregulation of genes encoding DAHPS, EPSPS, and DHQS was accompanied by a substantial increase in tyrosine (~4-fold at 72 h), whereas MA showed only transient gene induction and minor changes in amino acid levels (**Figure 6**). This suggests that DP exhibits a compensatory activation of the shikimate pathway to overcome EPSPS inhibition, maintaining metabolic flux toward key downstream pathways—a feature previously associated with stress-tolerant genotypes (Tohge et al., 2013).

Downstream of the shikimate pathway, the phenylpropanoid metabolism—which synthesizes lignin and flavonoids—was also distinctly modulated between the two cultivars. These compounds are essential for reinforcing cell walls and mitigating oxidative damage (Fraser and Chapple, 2011). In DP, there was rapid and sustained accumulation of phenylpropanoid intermediates such as caffeic acid, ferulic acid, and coniferyl alcohol (**Figure 7**). Notably, ferulic acid levels increased ~4-fold as early as 1 h in DP, suggesting early activation of lignin biosynthesis. Additionally, in the flavonoid branch, DP showed significant accumulation of naringenin chalcone and naringenin, with the latter rising nearly 3-fold at 24 h, unlike in MA. These metabolites are known to enhance antioxidant capacity and stress resilience (Agati et al., 2012). Collectively, the enhanced biosynthesis of lignin and flavonoids in DP likely contributes to its improved structural integrity and redox balance under glyphosate stress. These findings agree with previous studies showing that phenylpropanoid-derived metabolites play essential roles in herbicide tolerance (Yuan et al., 2022).

Together, our integrated transcriptomic and metabolomic analyses reveal a multifaceted resistance mechanism in the glyphosate-tolerant cultivar DP, involving precise transcriptional regulation, sustained activation of the shikimate pathway, and strategic rerouting of metabolic flux toward the biosynthesis of protective secondary metabolites, including lignin and flavonoids. These coordinated molecular and metabolic adjustments likely contribute to enhanced oxidative stress mitigation and cellular protection under glyphosate exposure. In contrast, the sensitive cultivar MA exhibited broader but less functionally focused responses, characterized by disrupted metabolic processes and limited transcriptional regulation, which may underlie its heightened susceptibility to glyphosate-induced damage. From an applied perspective, the pathways and metabolites identified in DP highlight potential targets for breeding glyphosate-tolerant potato cultivars. In particular, traits associated with metabolic reprogramming, redox homeostasis, and secondary metabolism could serve as valuable selection criteria or biomarkers in breeding programs aimed at improving herbicide tolerance. Nevertheless, this study has several limitations. The experiments were conducted under controlled conditions and focused on early to mid-term responses following glyphosate treatment, and functional validation of candidate genes and metabolites was not performed. Moreover, the analysis was limited to two cultivars, which may constrain the broader applicability of the findings.

Future research should therefore focus on validating the functional roles of key genes and metabolic pathways identified here, using genetic, biochemical, and physiological approaches, and on evaluating their contributions to glyphosate tolerance under field conditions. Expanding this integrative framework to a broader range of potato genotypes and incorporating additional regulatory layers, such as proteomics or epigenetic analyses, will further advance our understanding of glyphosate tolerance mechanisms and support the development of stress-resilient crop varieties.

## Data Availability

The datasets presented in this study can be found in online repositories. The names of the repository/repositories and accession number(s) can be found in the article/**Supplementary Material**.

## Supplementary material

Supplementary Table 1 Statistical summary of sequencing data quality control metrics.

Supplementary Table 2 Gene expression profiling across all experimental samples.

Supplementary Table 3 Differentially expressed genes (DEGs) identified in DP and MA cultivars.

Supplementary Table 4 Metabolite accumulation profiles showing differential regulation in DP and MA.

Supplementary Figure 1 GO enrichment bubble chart depicting biological process enrichment for DEGs in DP vs. MA.

Supplementary Figure 2 Comparative expression analysis of peroxidase (POD) genes in DP and MA cultivars. Heatmap visualization of temporal expression dynamics (1 h–120 h post-treatment).

Supplementary Figure 3 ABC transporter gene expression analysis. Differential expression patterns (fold change) between DP and MA under glyphosate stress.

Supplementary Figure 4 UDP-glycosyltransferase (UGT) gene expression heatmap. Cultivar-specific regulation of UGTs in response to herbicide treatment.
