# Supplementary figures and images for: Metabolic reprogramming and transcriptomic adaptation contribute to glyphosate resistance in potato cultivars
Source: Front Plant Sci. 2026 Feb 12;17:1757471. doi: 10.3389/fpls.2026.1757471 (PMC12935873; doi:10.3389/fpls.2026.1757471)

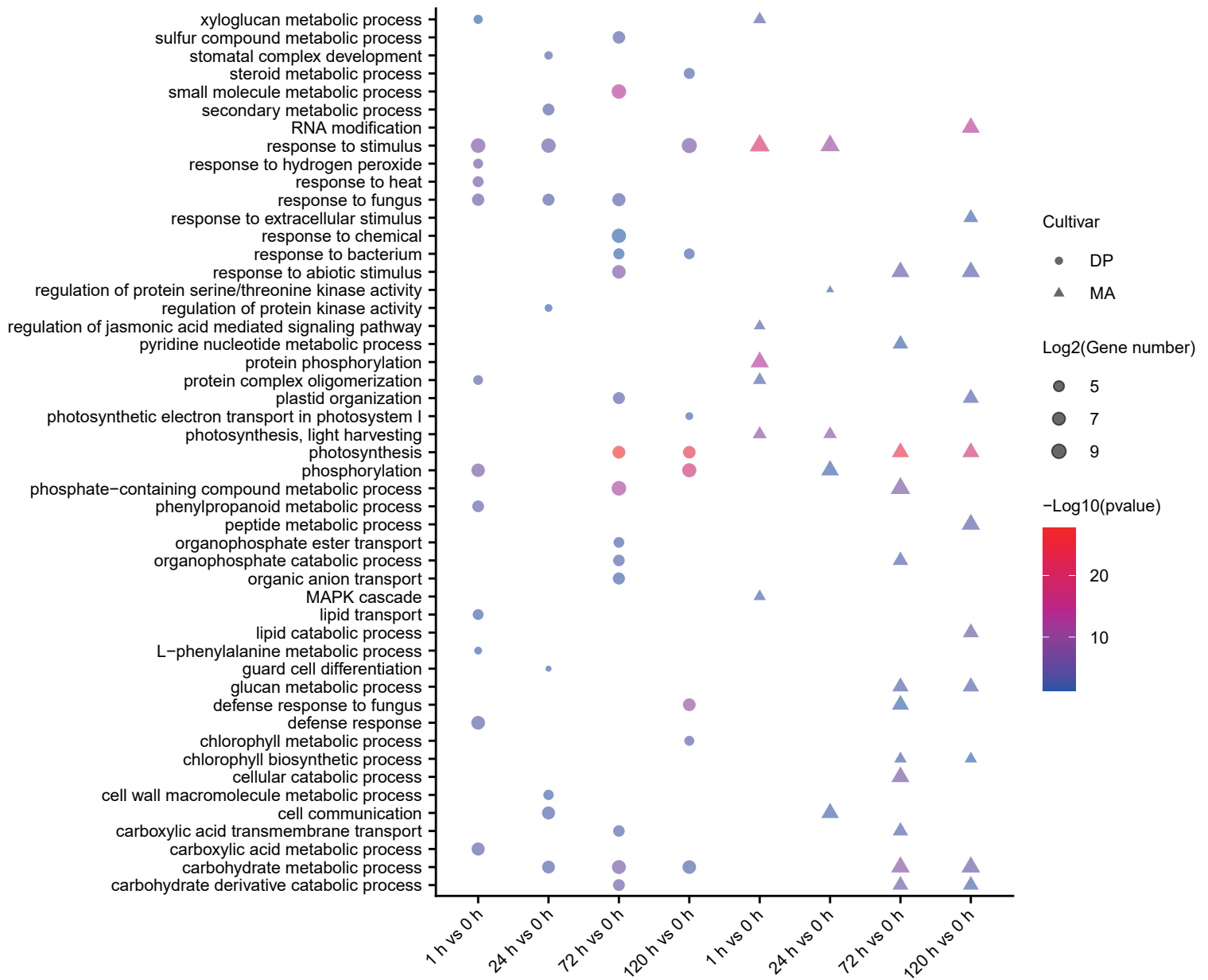

Supplement: Supplementary Figure 1 — GO enrichment bubble chart depicting biological process enrichment for DEGs in DP vs. MA. [file DataSheet1.pdf]

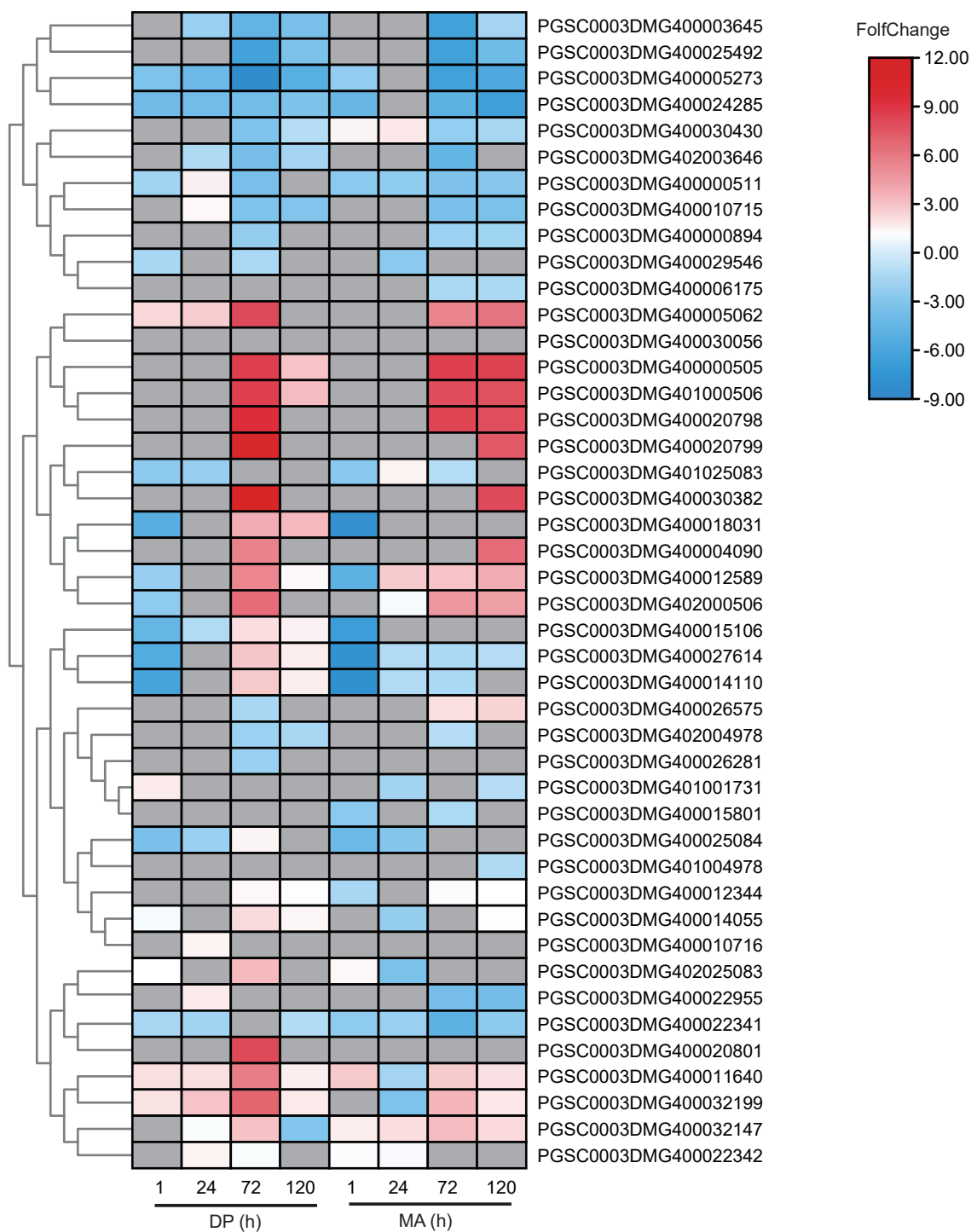

Supplement: Supplementary Figure 2 — Comparative expression analysis of peroxidase (POD) genes in DP and MA cultivars. Heatmap visualization of temporal expression dynamics (1 h–120 h post-treatment). [file DataSheet2.pdf]

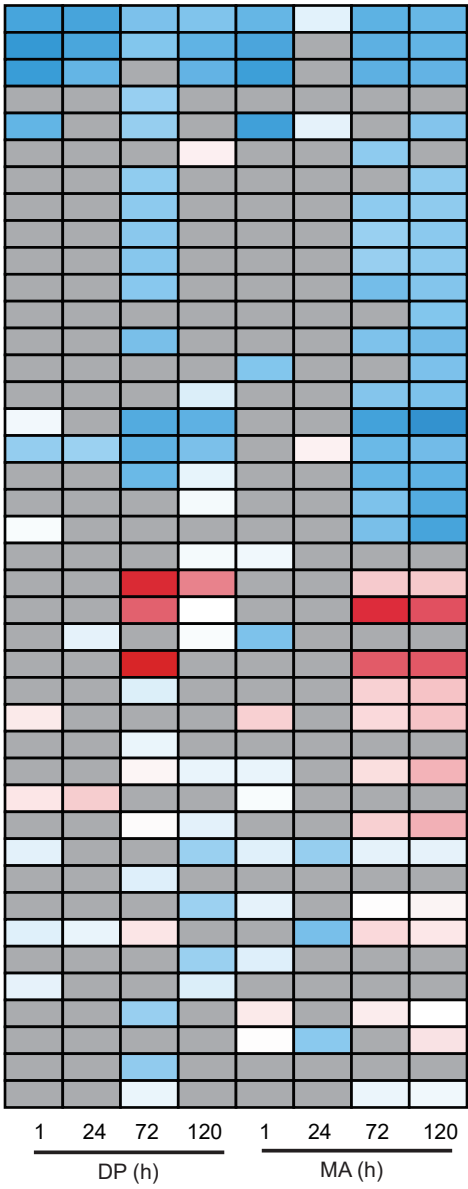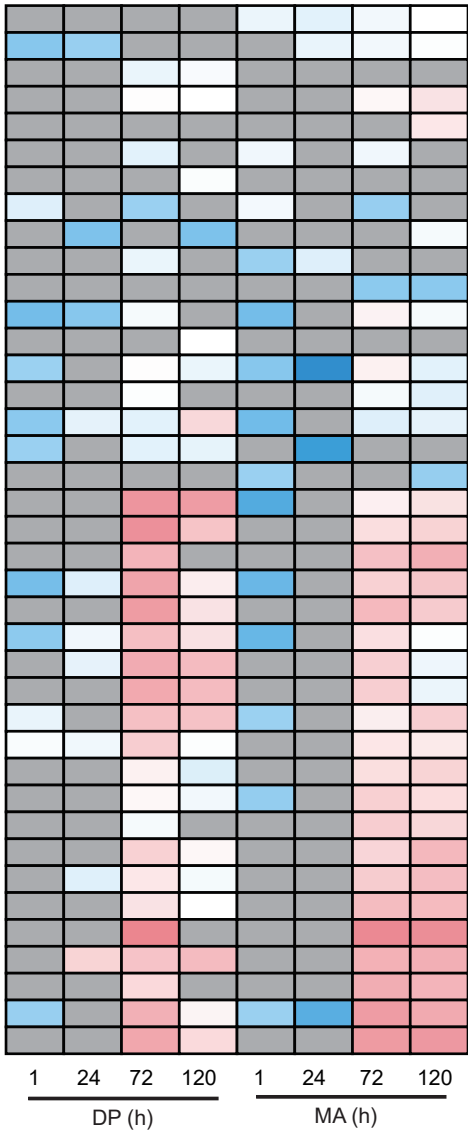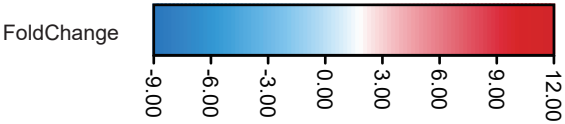

Supplement: Supplementary Figure 3 — ABC transporter gene expression analysis. Differential expression patterns (fold change) between DP and MA under glyphosate stress. [file DataSheet3.pdf]
