# Supplementary material for: Metabolic reprogramming and transcriptomic adaptation contribute to glyphosate resistance in potato cultivars
Source: Front Plant Sci. 2026 Feb 12;17:1757471. doi: 10.3389/fpls.2026.1757471 (PMC12935873; doi:10.3389/fpls.2026.1757471)
Supplement: Supplementary Figure 4 — UDP-glycosyltransferase (UGT) gene expression heatmap. Cultivar-specific regulation of UGTs in response to herbicide treatment. [file DataSheet4.pdf]

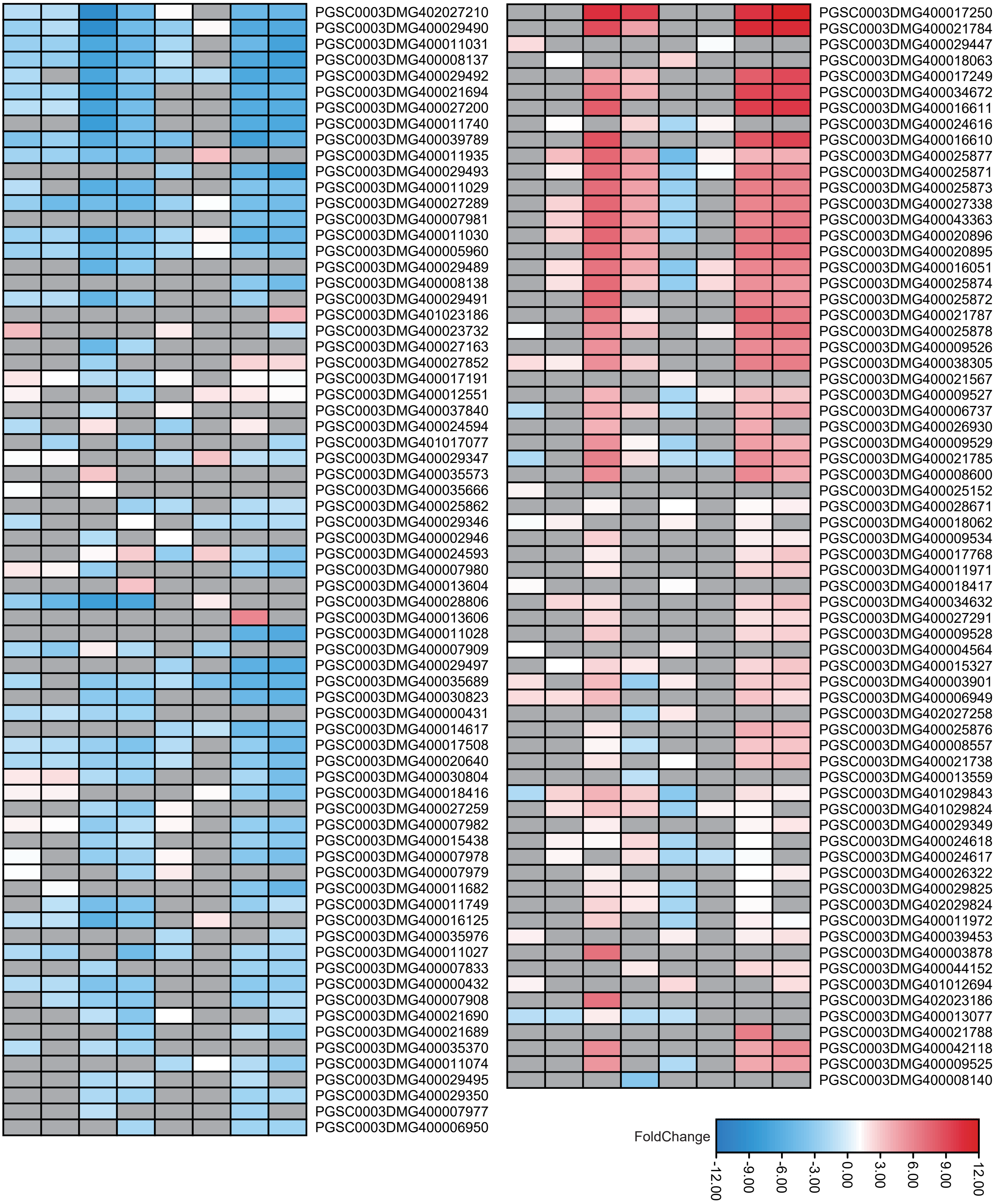
PGSC0003DMG402027210
PGSC0003DMG400029490
PGSC0003DMG400011031
PGSC0003DMG400008137
PGSC0003DMG400029492
PGSC0003DMG400021694
PGSC0003DMG400027200
PGSC0003DMG400011740
PGSC0003DMG400039789
PGSC0003DMG400011935
PGSC0003DMG400029493
PGSC0003DMG400011029
PGSC0003DMG400027289
PGSC0003DMG400007981
PGSC0003DMG400011030
PGSC0003DMG400005960
PGSC0003DMG400029489
PGSC0003DMG400008138
PGSC0003DMG400029491
PGSC0003DMG401023186
PGSC0003DMG400023732
PGSC0003DMG400027163
PGSC0003DMG400027852
PGSC0003DMG400017191
PGSC0003DMG400012551
PGSC0003DMG400037840
PGSC0003DMG400024594
PGSC0003DMG401017077
PGSC0003DMG400029347
PGSC0003DMG400035573
PGSC0003DMG400035666
PGSC0003DMG400025862
PGSC0003DMG400029346
PGSC0003DMG400002946
PGSC0003DMG400024593
PGSC0003DMG400007980
PGSC0003DMG400013604
PGSC0003DMG400028806
PGSC0003DMG400013606
PGSC0003DMG400011028
PGSC0003DMG400007909
PGSC0003DMG400029497
PGSC0003DMG400035689
PGSC0003DMG400030823
PGSC0003DMG400000431
PGSC0003DMG400014617
PGSC0003DMG400017508
PGSC0003DMG400020640
PGSC0003DMG400030804
PGSC0003DMG400018416
PGSC0003DMG400027259
PGSC0003DMG400007982
PGSC0003DMG400015438
PGSC0003DMG400007978
PGSC0003DMG400007979
PGSC0003DMG400011682
PGSC0003DMG400011749
PGSC0003DMG400016125
PGSC0003DMG400035976
PGSC0003DMG400011027
PGSC0003DMG400007833
PGSC0003DMG400000432
PGSC0003DMG400007908
PGSC0003DMG400021690
PGSC0003DMG400021689
PGSC0003DMG400035370
PGSC0003DMG400011074
PGSC0003DMG400029495
PGSC0003DMG400029350
PGSC0003DMG400007977
PGSC0003DMG400006950
PGSC0003DMG400017250
PGSC0003DMG400021784
PGSC0003DMG400029447
PGSC0003DMG400018063
PGSC0003DMG400017249
PGSC0003DMG400034672
PGSC0003DMG400016611
PGSC0003DMG400024616
PGSC0003DMG400016610
PGSC0003DMG400025877
PGSC0003DMG400025871
PGSC0003DMG400025873
PGSC0003DMG400027338
PGSC0003DMG400043363
PGSC0003DMG400020896
PGSC0003DMG400020895
PGSC0003DMG400016051
PGSC0003DMG400025874
PGSC0003DMG400025872
PGSC0003DMG400021787
PGSC0003DMG400025878
PGSC0003DMG400009526
PGSC0003DMG400038305
PGSC0003DMG400021567
PGSC0003DMG400009527
PGSC0003DMG400006737
PGSC0003DMG400026930
PGSC0003DMG400009529
PGSC0003DMG400021785
PGSC0003DMG400008600
PGSC0003DMG400025152
PGSC0003DMG400028671
PGSC0003DMG400018062
PGSC0003DMG400009534
PGSC0003DMG400017768
PGSC0003DMG400011971
PGSC0003DMG400018417
PGSC0003DMG400034632
PGSC0003DMG400027291
PGSC0003DMG400009528
PGSC0003DMG400004564
PGSC0003DMG400015327
PGSC0003DMG400003901
PGSC0003DMG400006949
PGSC0003DMG402027258
PGSC0003DMG400025876
PGSC0003DMG400008557
PGSC0003DMG400021738
PGSC0003DMG400013559
PGSC0003DMG401029843
PGSC0003DMG401029824
PGSC0003DMG400029349
PGSC0003DMG400024618
PGSC0003DMG400024617
PGSC0003DMG400026322
PGSC0003DMG400029825
PGSC0003DMG402029824
PGSC0003DMG400011972
PGSC0003DMG400039453
PGSC0003DMG400003878
PGSC0003DMG400044152
PGSC0003DMG401012694
PGSC0003DMG402023186
PGSC0003DMG400013077
PGSC0003DMG400021788
PGSC0003DMG400042118
PGSC0003DMG400009525
PGSC0003DMG400008140
FoldChange
-12.00
-9.00
-6.00
-3.00
0.00
3.00
6.00
9.00
12.00
